# Buckysomes: New Nanocarriers for Anticancer Drugs

**DOI:** 10.1155/2013/390425

**Published:** 2013-02-28

**Authors:** Delia Danila, Eva Golunski, Ranga Partha, Madonna McManus, Tina Little, Jodie Conyers

**Affiliations:** ^1^Division of Cardiology, Department of Internal Medicine, The University of Texas Health Science Center at Houston, 1881 East Road, Houston, TX 77054, USA; ^2^Biomatrica, 5627 Oberlin Drive, San Diego, CA 92121, USA; ^3^Graduate Schools of Biomedical Sciences, The University of Texas Health Science Center at Houston, 6767 Bertner Ave, Houston, TX 77030, USA; ^4^Department of Anesthesiology, The University of Texas Health Science Center at Houston, 6431 Fannin Street, Houston, TX 7703, USA; ^5^Casscells & Associates, LLC, 7111 Davis Court, McLean, VA 22101, USA

## Abstract

Buckysomes, liposome-like vesicles comprised of dendritic C60 subunits that self-assemble into unilamellar vesicles, are unique nanovectors that have utility in drug delivery. We have prepared paclitaxel-embedded buckysomes (PEBs) and examined biodistriubition profiles with commercially available formulations of the drug. As compared to Abraxane, an albumin-bound formulation of paclitaxel, PEBs showed higher tissue accumulation in the liver and the kidney at 45 and 60 minutes and in the lungs at 30 minutes, making them suitable drug-delivery carriers for short-term therapy to the mentioned organs. These buckysomes can be further functionalized to specifically deliver paclitaxel to the tumor site.

## 1. Introduction

Paclitaxel is a very potent anticancer drug that has been used since 1967 to treat breast, ovarian, and lung cancer, as well as advanced forms of Kaposi's sarcoma [1]. Paclitaxel is a hydrophobic drug (solubility ~0.3 *μ*g/mL) [2] that was previously administered via dissolution in Cremophor EL (polyoxyethylated castor oil) and dehydrated alcohol. However, the castor oil proved to be toxic [3, 4], so other alternatives for paclitaxel delivery were investigated, such as the use of nanoscale carrier vectors [2, 5, 6]. Among the different types of nanocarriers, liposomes have received a lot of attention and have proven to be very good carriers of drugs and/or contrast agents [7].

**Figure 1:**
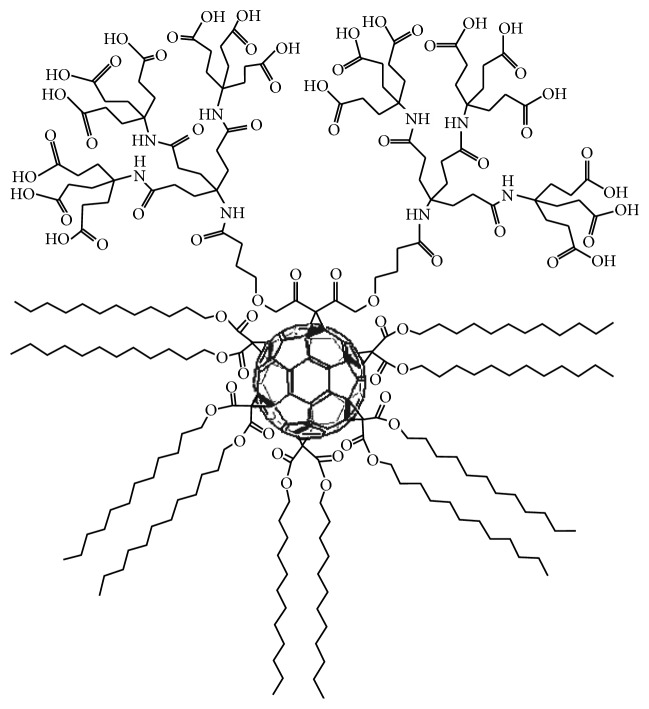
Chemical structure of the amphiphilic fullerene monomer AF-1. AF-1 has a molecular weight of 5022, a length of 3.5 nm, and a width of 2.3 nm.

Paclitaxel-embedded buckysomes have the potential for delivering the hydrophobic drug directly to tumor sites. The buckysomes are self-assembled structures of the monomer AF-1, a modified water soluble fullerene that contains 18 carboxylic acid groups and 5 didodecyl malonate chains around the C60 fullerene (Figure 1). This unique functionalization permits the creation of buckysome structures that might offer advantages over traditional phospholipid liposomes as nanovectors. The buckysomes are formulated so they have a dense hydrophobic region, making them ideal for embedding hydrophobic molecules, such as paclitaxel. Liposomes are mostly suitable for carrying a hydrophilic payload in their hydrophilic compartment and less suitable for hydrophobic materials. Another advantage of using buckysomes is the lack of organic solvents during their formation steps as well as their ease of preparation. 

Previous studies in our group report the synthesis and characterization of buckysome nanovectors for hydrophobic molecule delivery [8]. The therapeutic efficacy of paclitaxel-embedded buckysomes was tested *in vitro *and compared to that of Abraxane, a commercially available albumin-bound formulation of paclitaxel. The paclitaxel-embedded buckysomes demonstrated a similar efficacy to that of Abraxane when tested on MCF-7 breast cancer cells, where we observed mitigated cell division at 48 and 72 hours [9]. These results opened the door to study the clearance and biodistribution of paclitaxel-embedded buckysomes in an animal model and study them as possible drug carriers *in vivo*. 

Thus, in this study we evaluated the clearance and biodistribution of paclitaxel-embedded buckysomes in ICR mice and compared the results to those of Abraxane.

## 2. Material and Methods

### 2.1. Materials

AF-1 was synthesized in Dr. Hirsch's lab (Germany) [10], citrate buffer was purchased from Sigma-Aldrich, paclitaxel was purchased from VWR, and Abraxane was obtained from Abraxis Oncology.

### 2.2. Synthesis of Paclitaxel-Embedded Buckysomes (PEBs)

Empty buckysomes preparation was described in a previous paper published by our group [9]. Briefly, to create buckysomes with a dense, hydrophobic interior, AF-1 (1.5 mg/mL) was hydrated in 10 mM citrate buffer (pH 7.4) for 10 min at 70°C. During this process, the mixture was subjected to four vortex cycles for 30 s. The four vortex cycles were separated by equal time intervals. To prepare paclitaxel-embedded buckysomes (PEBs), 0.6 mg/mL paclitaxel was sonicated (Cole-Parmer sonicator (model no. 08849-00), Vernon Hills, IL) for 1 min in 10 mM citrate buffer (pH 7.4). AF-1 was quickly added to this solution at a concentration of 1.5 mg/mL, and PEBs were prepared from the resulting solution using the method described previously. 

### 2.3. Pharmacokinetic and Biodistribution Study

The animal studies were performed on female ICR mice (25–30 g) ordered from Harlan, and they were housed at the University of Texas Health Science Center at Houston (UTHSCH). The animal protocol was approved by the UTHSCH Center for Laboratory Animal Medicine and Care (protocol no.: HSC-AWC-08-137, “Preclinical Development Studies on Paclitaxel-Embedded Fullerene Nanoparticles”); experiments were performed in accordance with institutional guidelines, and all efforts were made to minimize suffering.

The mice were divided into 4 groups: (1) mice injected with saline (250 *μ*L) (control), (2) mice injected with Abraxane (250 *μ*L, 0.2 mg/mL) (positive control), (3) mice injected with empty buckysomes (250 *μ*L, 2 mg/mL AF-1), (4) mice injected with paclitaxel-embedded buckysomes (250 *μ*L, 0.2 mg/mL paclitaxel, and 2 mg/mL AF-1). Mice were injected through the tail vain with the various materials and sacrificed at 15, 30, 45, 60, and 180 minutes. Immediately after sacrifice, at every time point, blood along with kidney, liver, spleen, lungs, heart, and brain were collected and stored at −80°C before analysis. The amount of AF-1 and/or paclitaxel present in each organ was quantified by HPLC after extraction as presented in the Supplementary Material section (see Supplementary Material available online at http://dx.doi.org/10.1155/2013/390425). Five mice per time point were analyzed.

## 3. Statistical Analysis

The unpaired *t*-test was performed to establish comparison between the control and the paclitaxel groups in PEB.

## 4. Results and Discussion

The biodistribution of Abraxane, paclitaxel in PEB, AF-1 in empty buckysomes, and AF-1 in PEB in major tissues was monitored for 180 minutes after IV injection in ICR mice. The extraction efficiencies for Abraxane were 52, 37, 26, 18, 24, 35, and 37 percent for blood, liver, spleen, heart, kidney, lung, and brain, respectively. As for the paclitaxel in PEB, the extraction efficiencies were 48, 18, 36, 54, 35, 40, and 24 percent for blood, liver, spleen, heart, kidney, lung, and brain, respectively. The extraction efficiencies were used for data corrections.

**Figure 2:**
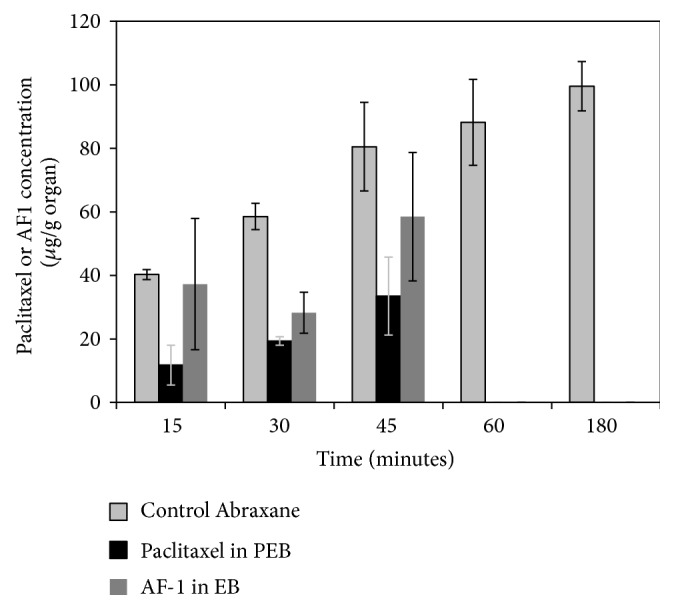
Concentration of Abraxane, paclitaxel in paclitaxel-embedded-buckysomes, and AF-1 in empty buckysomes in blood after tail vain injection of a single 0.2 mg/mL dose Abraxane/paclitaxel and 2 mg/mL AF-1. Concentration is expressed in *μ*g drug/g organ. The results are expressed as the mean ± S.E. (*n* = 3 for Abraxane, *n* = 5 for PEBs, *n* = 5 for EBs).

**Figure 3:**
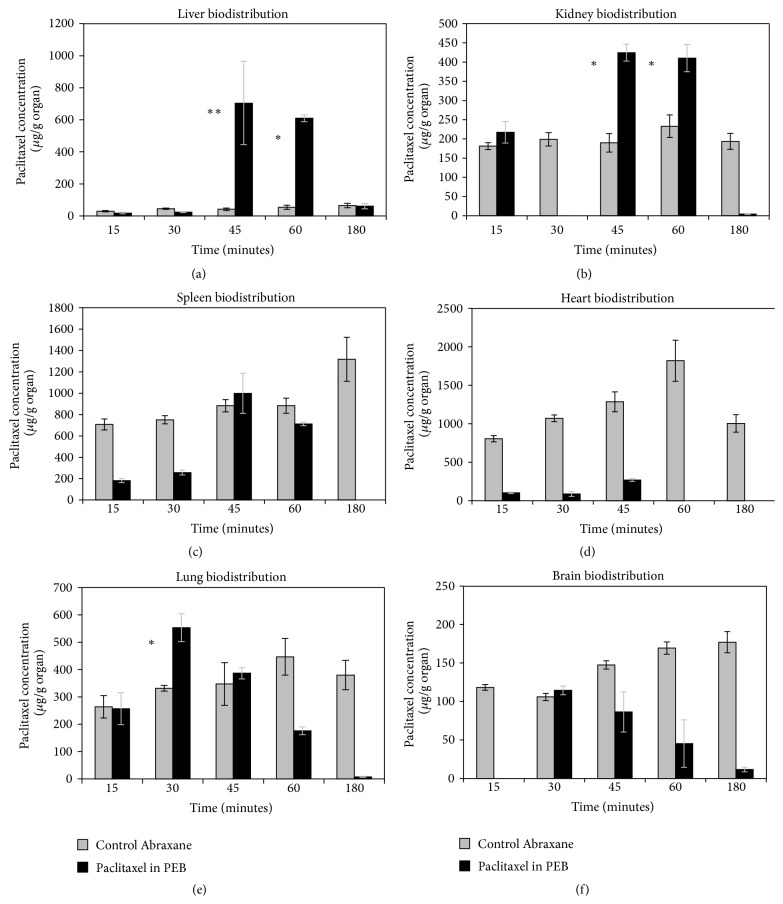
Organ biodistribution of Abraxane and paclitaxel in paclitaxel-embedded buckysomes at 15, 30, 45, 60, and 180 minutes after tail vein injection in ICR mice. The dose administered was 0.2 mg Abraxane/paclitaxel/mL. The results are expressed as the mean ± S.E. (*n* = 3 for Abraxane, *n* = 5 for PEB). ^*^ *P* < 0.0001 versus control; ^**^ *P* < 0.05 versus control.

The distribution profile for plasma after tail vein injection of a single dose of Abraxane, paclitaxel in PEB (0.2 mg/mL paclitaxel + 2 mg/mL AF-1), or AF-1 in EB (2 mg/mL AF-1) is shown in Figure 2. Concentration is expressed in *μ*g drug/g organ. The results are expressed as the mean ± S.E. (*n* = 3 for Abraxane, *n* = 5 for PEBs, *n* = 5 for EBs). Abraxane exhibits a longer circulation time as compared to paclitaxel in PEB. After 180 minutes, there is still measurable amount of Abraxane in circulation. However, there is no measurable amount of paclitaxel in circulation after 180 minutes. In order to study if paclitaxel was still associated with the buckysomes after tail vein injection, the distribution profile for plasma was compared between paclitaxel in PEB (0.2 mg/mL paclitaxel + 2 mg/mL AF-1) and AF-1 in EB (2 mg/mL AF-1). The results are presented in Figure 2 and clearly demonstrate that paclitaxel and AF-1 have the same clearance profile, indicating that paclitaxel was encapsulated in buckysomes. The biodistribution profiles for liver, kidney, spleen, heart, lung, and brain at 15, 30, 45, 60, and 180 minutes after tail vein injection in ICR mice are shown in Figure 3. The dose administered was 0.2 mg/mL Abraxane/paclitaxel. The results are expressed as the mean ± S.E. (*n* = 3 for Abraxane, *n* = 5 for PEB, ^*^ *P* < 0.0001 versus control; ^**^ *P* < 0.05 versus control). Our results indicated that the buckysomes are cleared rapidly via hepatic and renal pathways (Figures 3(a) and 3(b)). Paclitaxel reaches high concentrations at forty-five minutes and sixty minutes after IV injections of paclitaxel-embedded buckysomes in liver and kidneys. Also, the buckysomes exhibit a high peak of paclitaxel in the lungs (Figure 3(e)) at 30 minutes after injection. The biodistribution profiles for the other organs and for the other time points indicate that Abraxane was a better long-term therapy drug. The concentration of paclitaxel in PEB was lower in plasma (at all-time points), kidney (at 30 min, and 180 min), spleen (at 15, 30, 60, and 180 min), heart (at all-time points), lung (at 60 and 180 min), and brain (at 15, 45, 60, and 180 min) compared to that in the control. We also get very similar distribution profiles in the previously mentioned organs for both AF-1 in EB and paclitaxel in PEB (results presented in Supplementary Material), indicating that paclitaxel is strongly associated with the buckysomes and conforming that the buckysomes are mostly suitable as a short-term delivery carrier.

## 5. Conclusions

Paclitaxel-embedded buckysomes were previously synthesized and characterized *in vitro* in our lab and seemed to be very promising novel delivery vehicles. In the present study, paclitaxel-embedded buckysomes were characterized in terms of their clearance and biodistribution profile, and the results were compared to those for Abraxane. PEBs did not exhibit a high plasma concentration, and they were cleared in about 60 minutes by liver and kidneys. The administration of Abraxane was associated with higher blood concentrations as compared to that of paclitaxel-embedded buckysomes with measurable amounts of paclitaxel in Abraxane at 180 minutes. The biodistribution of paclitaxel in PEBs for other organs also demonstrated that, overall, Abraxane proved to be a better long-circulating drug. However, PEBs showed higher tissue accumulation in the liver and kidney at 45 and 60 minutes and in the lungs at 30 minutes, making them suitable drug-delivery carriers for short-term therapy to the mentioned organs. Our results also clearly indicate that paclitaxel was successfully encapsulated in the hydrophobic interior of buckysomes as the blood clearance profile as well as the organ biodistribution for paclitaxel in paclitaxel-embedded buckysomes is similar to that of AF-1 in empty buckysomes. Future studies will focus on targeting the paclitaxel-embedded buckysomes to subcutaneously implanted tumors and study their efficacy as compared to the commercially available Abraxane. 

## Supplementary Material

A detailed description of the methods used for the extraction of Abraxane/Paclitaxel/AF-1 from various organs and the HPLC analysis of Abraxane, AF-1, and paclitaxel after extraction from organs is presented in the Supplementary Material. Details on abraxane/paclitaxel extraction efficiency measurements are also provided.
The organ biodistribution of paclitaxel in paclitaxel-embedded buckysomes and AF-1 in empty buckysomes at 15, 30, 45, 60, and 180 minutes after tail vein injection in ICR mice is presented in the Supplementary Material in order to support the conclusion that paclitaxel was successfully encapsulated in the hydrophobic interior of buckysomes.
