# Supplementary material for: Buckysomes: New Nanocarriers for Anticancer Drugs
Source: J Pharm (Cairo). 2013 Feb 28;2013:390425. doi: 10.1155/2013/390425 (PMC4600851; doi:10.1155/2013/390425)
Supplement: Supplementary file 1 — A detailed description of the methods used for the extraction of Abraxane/Paclitaxel/AF-1 from various organs and the HPLC analysis of Abraxane, AF-1, and paclitaxel after extraction from organs is presented in the Supplementary Material. Details on abraxane/paclitaxel extraction efficiency measurements are also provided. The organ biodistribution of paclitaxel in paclitaxel-embedded buckysomes and AF-1 in empty buckysomes at 15, 30, 45, 60, and 180 minutes after tail vein injection in ICR mice is presented in the Supplementary Material in order to support the conclusion that paclitaxel was successfully encapsulated in the hydrophobic interior of buckysomes. [file 390425.f1.docx]

***Supplementary Materials:***

*Extraction of Abraxane/Paclitaxel/AF-1:*

For the extraction of Abraxane, the organs were weighed, cut into small pieces, mixed with ethylacetate and homogenized for 1 minute using a mini-bead beater (Biospec Products, Model 2412PS-12W-B30). The samples were then centrifuged for 10 minutes (1850 rcf) and the supernatant was transferred to clean centrifuge tubes. Ethylacetate was evaporated on a heat block (70 °C) and the pellet was reconstituted in 250 μl methanol : acetonitrile (2:1 v/v). The samples were centrifuged for 10 minutes (1850 rcf) and the supernatant transferred to HPLC inserts for analysis. For the extraction of AF-1, the organs were weighed, cut into small pieces, mixed with chloroform and homogenized for 1 minute using a mini-bead beater (Biospec Products, Model 2412PS-12W-B30). Because of the fact that the organs solidified after treatment with chloroform, they were transferred with a spatula to 15 ml Falcon tubes and more chloroform was added. The samples were then centrifuged for 10 minutes (1850 rcf) and the supernatant was transferred to clean centrifuge tubes. Chloroform was evaporated overnight, the pellet was reconstituted in 500 μl ethanol (200 proof), and sonicated for 10 minutes. The samples were centrifuged for 10 minutes (1850 rcf) and the supernatant transferred to HPLC inserts for analysis. For extraction of both AF-1 and paclitaxel in PEBs, the organs were weighed, cut into small pieces, mixed with chloroform:ethylacetate (1:1, v/v) and homogenized for 1 minute using a mini-bead beater (Biospec Products, Model 2412PS-12W-B30). Because of the fact that the organs solidified after treatment with chloroform:ethylacetate, they were transferred with a spatula to 15 ml Falcon tubes and more solvent mixture was added. The samples were then centrifuged for 10 minutes (1850 rcf) and the supernatant was transferred to clean centrifuge tubes. Solvent mixture was evaporated overnight, the pellet was reconstituted in 500 μl ethanol (200 proof), and sonicated for 10 minutes. The samples were centrifuged for 10 minutes (1850 rcf) and the supernatant transferred to HPLC inserts for analysis.

*HPLC analysis (equipment and mobile phase):*

Abraxane, AF-1, and paclitaxel were quantified by HPLC after extraction from organs. The equipment consisted of a Waters HPLC (Waters, Milford, Massachusetts) system equipped with an analytical column (4.6x75 mm column) packed with C18 (3.5 μm).

For the analysis of paclitaxel, the mobile phase consisted of a mixture of water and acetonitrile (30:70, v/v). The separation was done isocratically with a flow rate of 1 ml/min. 50 μl samples were injected and the typical pressure was approximately 1700 psi. For detection, a photodiode array detector (Waters 2996) was used at an absorbance wavelength of 254 nm.

For the analysis of AF-1, the mobile phase consisted of a mixture of water and acetonitrile (76:24, v/v). The separation was done isocratically with a flow rate of 1 ml/min. 50 μl samples were injected and the typical pressure was approximately 1700 psi.

For abraxane/paclitaxel extraction efficiency measurements, a known amount of drug was injected directly into tissue, the drug was extracted and the amount of drug extracted was compared to the initial amount of abraxane/paclitaxel injected. The extraction efficiency was calculated as being:

% efficiency = [amount of drug after extraction)/(amount of drug added to tissue)]*100

The %efficiency was calculated for each organ and the value was used to calculate the final concentrations of drugs in the tissue.

Figure 1. Organ biodistribution of paclitaxel in paclitaxel-embedded buckysomes (Figure 1A) and AF-1 in empty buckysomes (Figure 1B) at 15, 30, 45, 60, and 180 minutes after tail vein injection in ICR mice. The dose administered was 0.2 mg/ml paclitaxel + 2 mg/ml AF-1 in PEB and 2 mg/ml AF-1 in EB. The results are expressed as the mean ±S.E. (n=5 for paclitaxel in PEB, n=5 for AF-1 in EB).

(A)

(B)
